# Draft Genomic Analysis of *Pseudomonas* sp. Strain OA3, a Potential Plant Growth-Promoting Rhizospheric Bacterium

**DOI:** 10.1128/MRA.00029-21

**Published:** 2021-03-18

**Authors:** Olubukola Oluranti Babalola, Oluwaseun Adeyinka Fasusi, Adenike Eunice Amoo, Ayansina Segun Ayangbenro

**Affiliations:** a Food Security and Safety Niche, Faculty of Natural and Agricultural Science, North-West University, Mmabatho, South Africa; University of Arizona

## Abstract

The genome analysis of the plant growth-promoting rhizospheric *Pseudomonas* sp. strain OA3, isolated from maize in North West Province, South Africa, is reported in this study. *Pseudomonas* sp. strain OA3 exhibits plant growth-promoting ability by enhancing maize and soybean growth.

## ANNOUNCEMENT

Globally, there has been an increase in population, which demands an intensification of food production. The application of chemical fertilizers in promoting plant growth, however, impairs human health and causes environmental pollution (1). To circumvent the challenges attributed to the use of chemical fertilizers, eco-friendly approaches are needed. The application of beneficial soil microorganisms for use as biofertilizers in promoting plant growth has been reported as one such method (2, 3). Thus, a rhizospheric bacterium was isolated from 10 g of maize rhizospheric soil from North-West University Agricultural Research Farm, South Africa (25°47′25.24056″S, 25°37′8.17464″E). Serial dilution was conducted, and a pure culture of the isolate was obtained by streaking it onto sterile Luria Bertani (LB) agar incubated at 28°C for 24 h. *Pseudomonas* sp. strain OA3 could potentially be used for plant growth promotion because it tested positive for *in vitro* plant growth-promoting tests such as phosphate solubilization (4), siderophore production (5), and the production of indole-3-acetic acid (6). The pure strain was kept on an agar slant for further use. DNA from the pure culture on solid agar was extracted using a soil microbe extraction kit (Zymo Research, USA). The genome sequence of *Pseudomonas* sp. strain OA3 was obtained using the Illumina MiSeq platform at the Agricultural Research Council Biotechnology Platform (ARC-BTP; Pretoria, South Africa).

The Illumina Nextera DNA Flex library preparation kit was used for the library preparation. The sequence reads generated using the MiSeq platform were in 2 × 250-bp format and were visualized using KBase (7). The raw data obtained from the sequencing run were 44,526,256 bp. The quality of the reads was checked using Fast QC version 1.0.4 (8). The low-quality reads and adapter regions from the data were filtered using Trimmomatic version 1.2.14 (9). The genome assembly was constructed using SPAdes version 1.2.4 (10), yielding a total genome assembly size of 5,473,200 bp with 2,410 contigs, a GC content of 62.09%, an *N*_50_ value of 3,046 bp, and an *L*_50_ value of 564. Default parameters were used for all software unless otherwise stated. Annotation for the prediction of gene functions was performed using the publicly available NCBI Prokaryotic Genome Annotation Pipeline (PGAP) (11). The result revealed 3,924 genes and 3,898 coding DNA sequences (CDSs) in total, 86 pseudogenes, and 49 noncoding sequences (26 rRNAs, 21 tRNAs, and 2 noncoding RNAs [ncRNAs]).

### Data availability.

The genome sequence of *Pseudomonas* sp. strain OA3 has been deposited at DDBJ/ENA/GenBank under the accession number JAEMOM000000000. The version described in this paper is version JAEMOM010000000. The raw reads are available under the BioProject accession number PRJNA683768 and the BioSample number SAMN17036469; the sequence data obtained in this work have been deposited in the NCBI Sequence Read Archive under the accession number SRX9654998.
